# Supplementary figures and images for: Human parvovirus B19 interacts with globoside under acidic conditions as an essential step in endocytic trafficking
Source: PLoS Pathog. 2021 Apr 20;17(4):e1009434. doi: 10.1371/journal.ppat.1009434 (PMC8087101; doi:10.1371/journal.ppat.1009434)

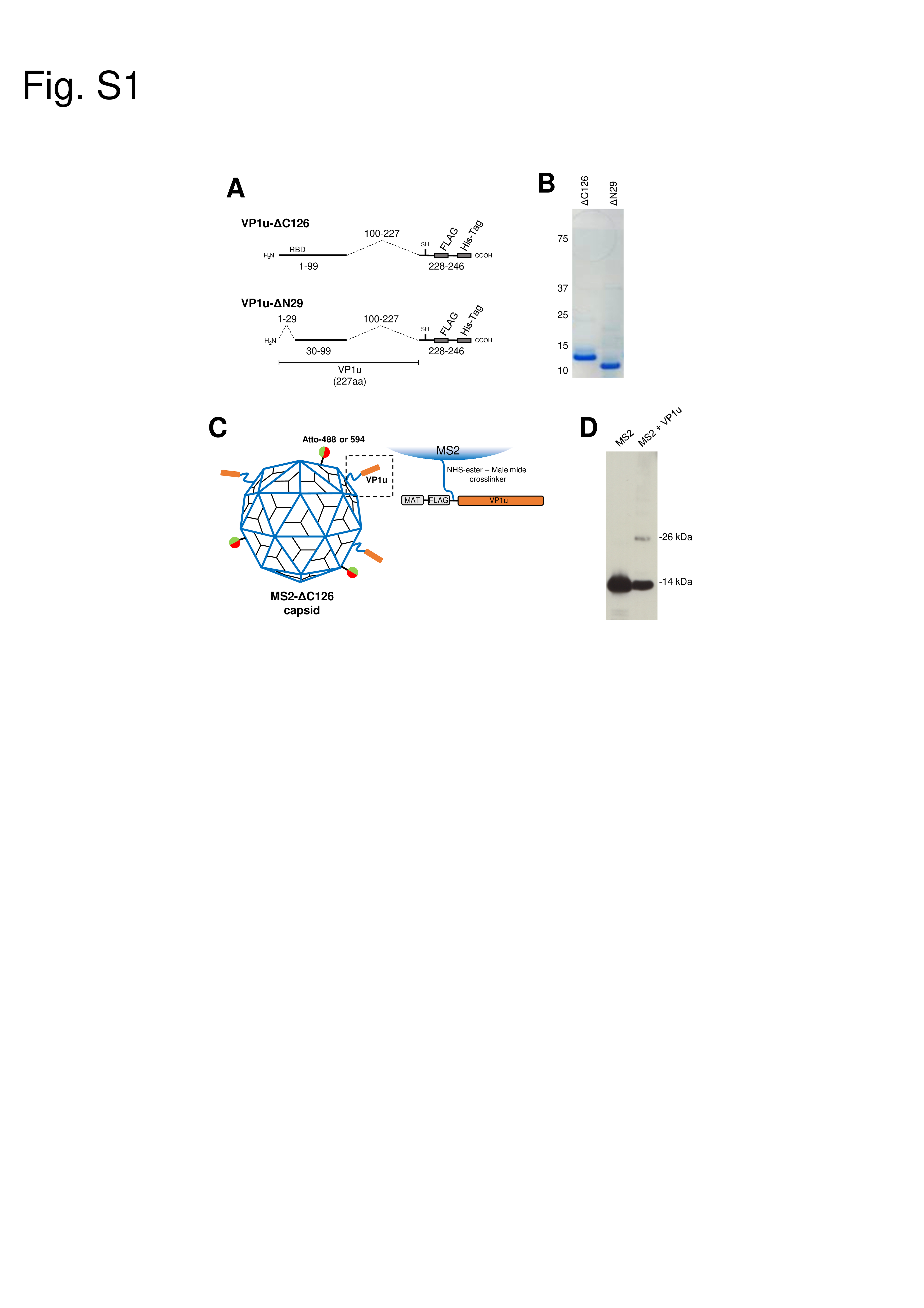

Supplement: S1 Fig — (A) Schematic depiction of the functional (ΔC126) and non-functional (ΔN29) recombinant VP1u constructs. (B) SDS-PAGE of purified recombinant VP1u constructs under reducing conditions. (C) Schematic depiction of an MS2 particle showing Atto fluorophores and VP1u constructs incorporated on the capsid surface. (D) Crosslinking between recombinant MS2 capsid proteins and VP1u constructs was verified by Western blot using an anti-MS2 antibody. (TIF) [file ppat.1009434.s001.tif]

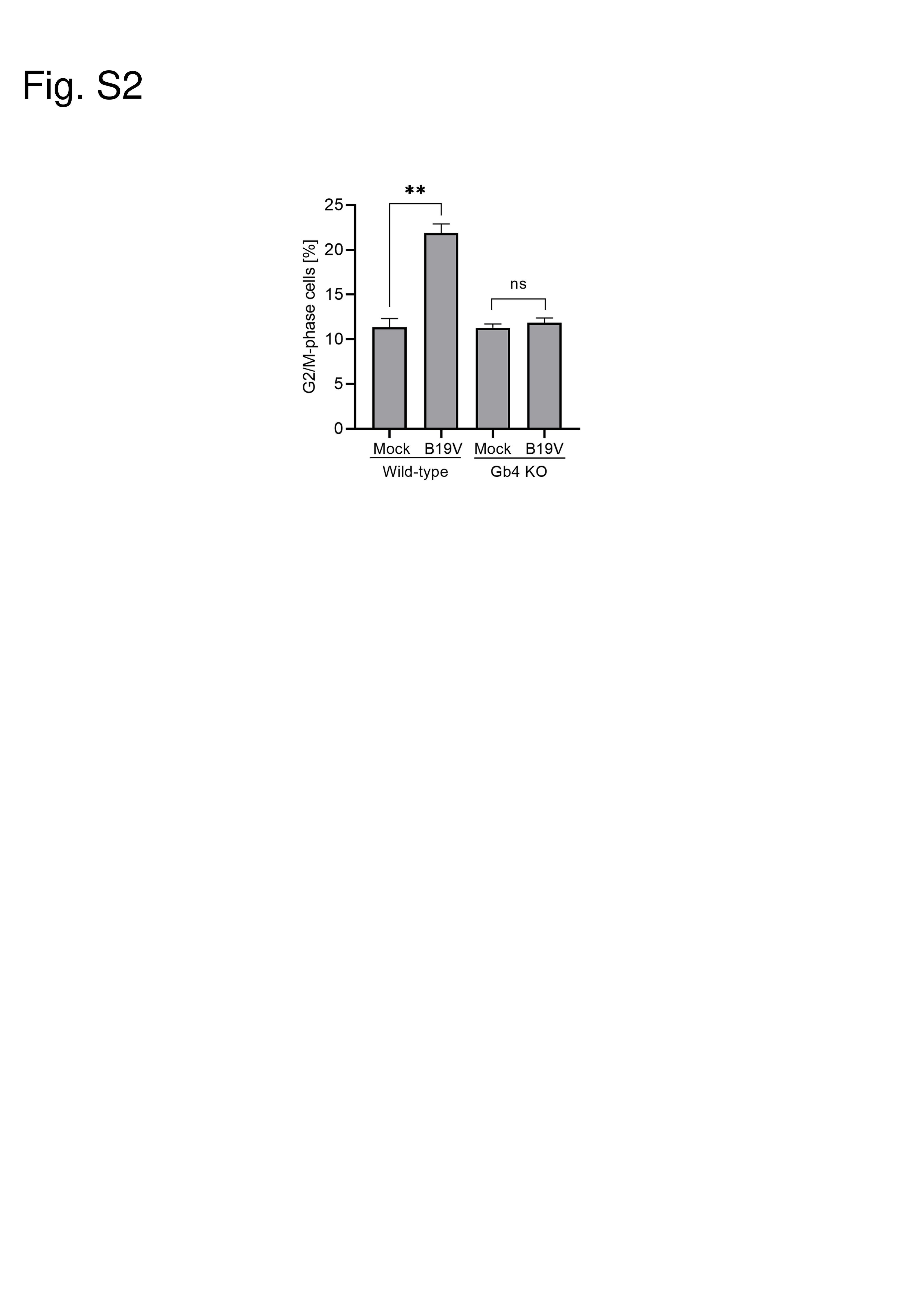

Supplement: S2 Fig — Cells were fixed 3d pi and cellular DNA was stained with DAPI. Cell cycle progression was analyzed using flow cytometry. The results are presented as the mean ± SD of three independent experiments. **, p<0.01; ns, not significant. (TIF) [file ppat.1009434.s002.tif]

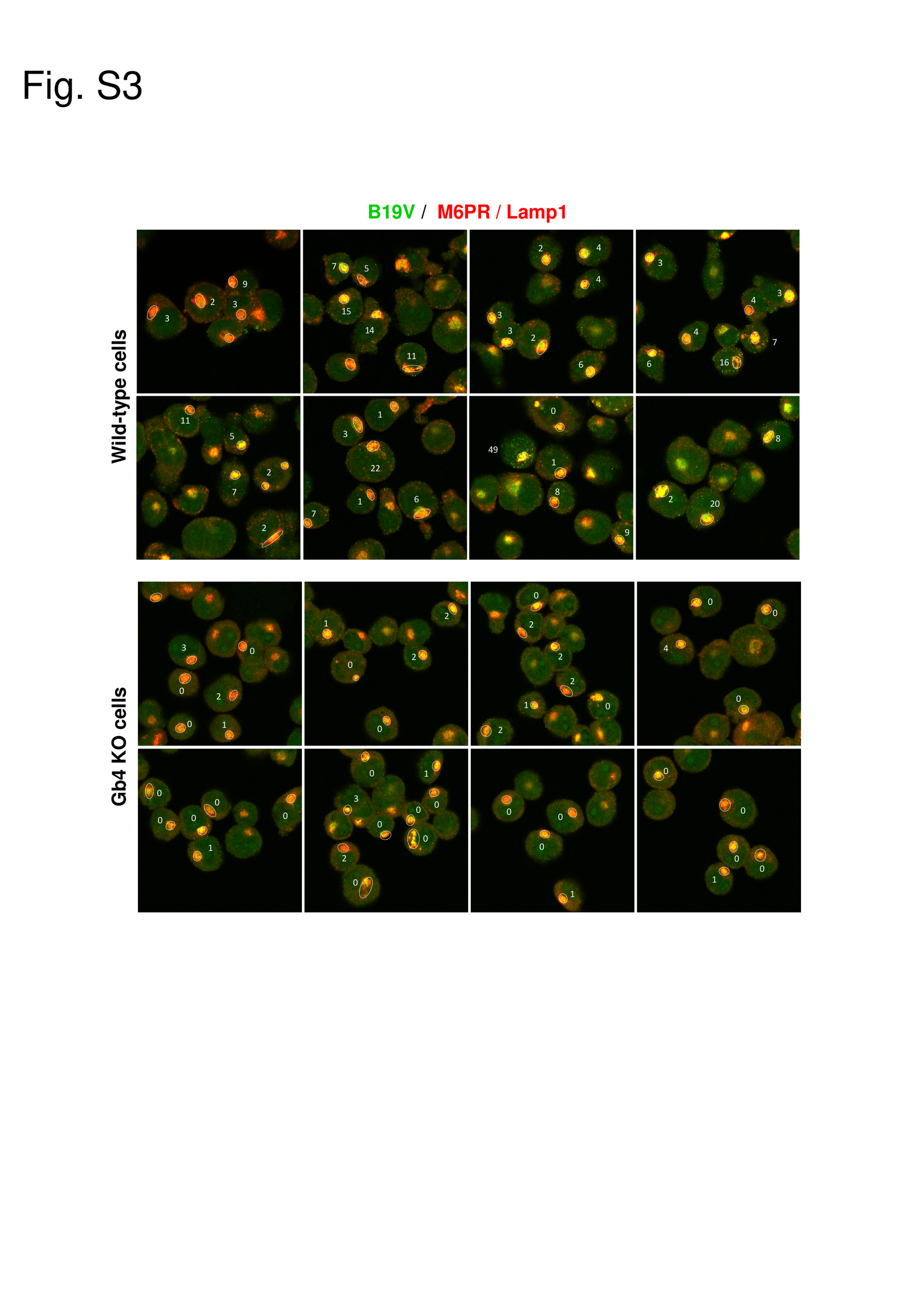

Supplement: S3 Fig — Cells exhibiting perinuclear cluster of endosomes in focus (encircled by a dotted line) were selected for analysis. Distinct, clearly visible fluorescent spots (B19V capsids; green) not colocalizing with endocytic markers (red) were counted. (TIF) [file ppat.1009434.s003.tif]

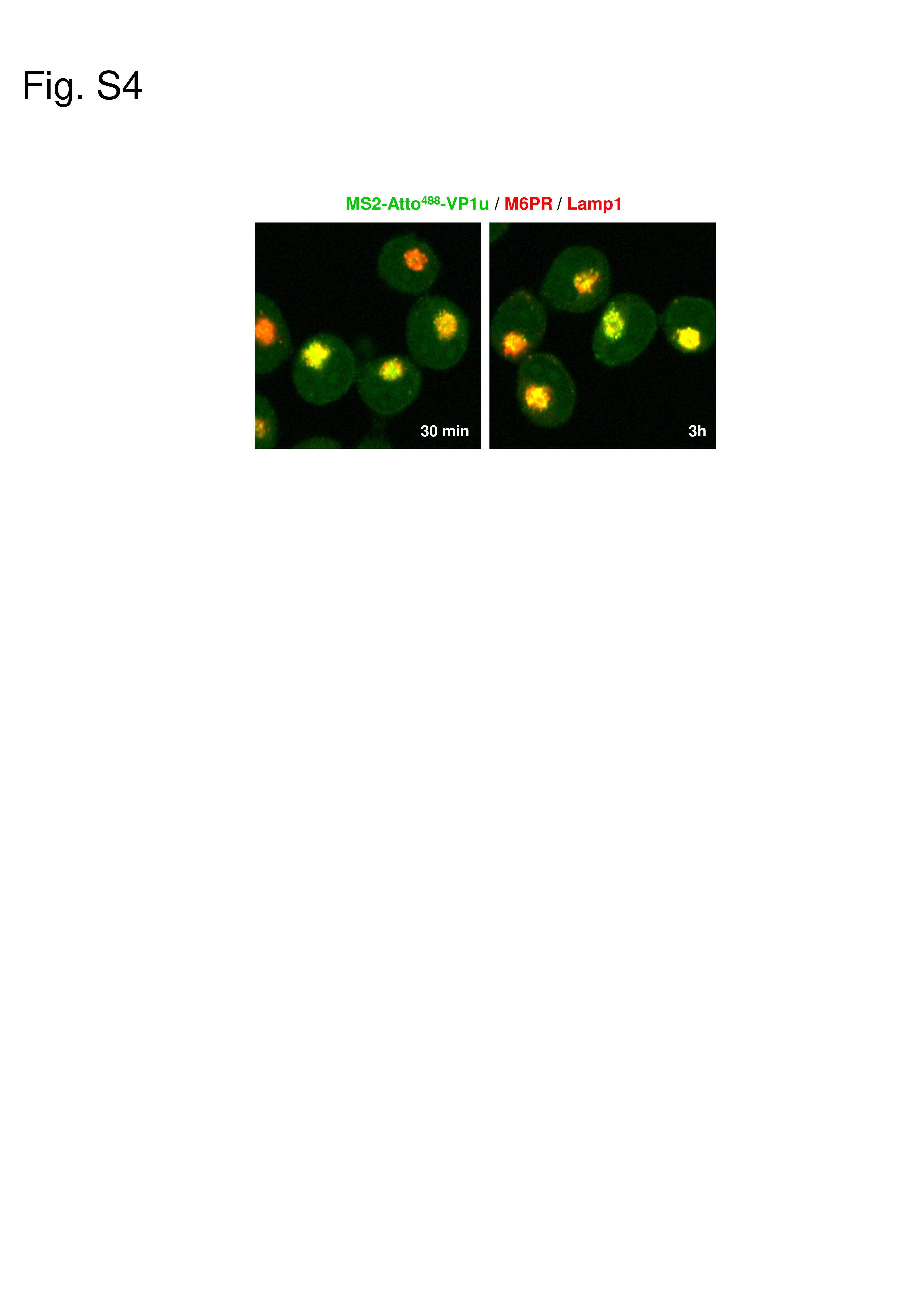

Supplement: S4 Fig — UT7/Epo cells (3x10^5^) were incubated with 2 μl Atto 488-labeled MS2-VP1u at 4°C for 1h, washed and further incubated at 37°C for 30 min and 3h. Cells were fixed and labeled with antibodies against late endosomes (M6PR) and lysosomes (Lamp1) and visualized under confocal microscopy. (TIF) [file ppat.1009434.s004.tif]

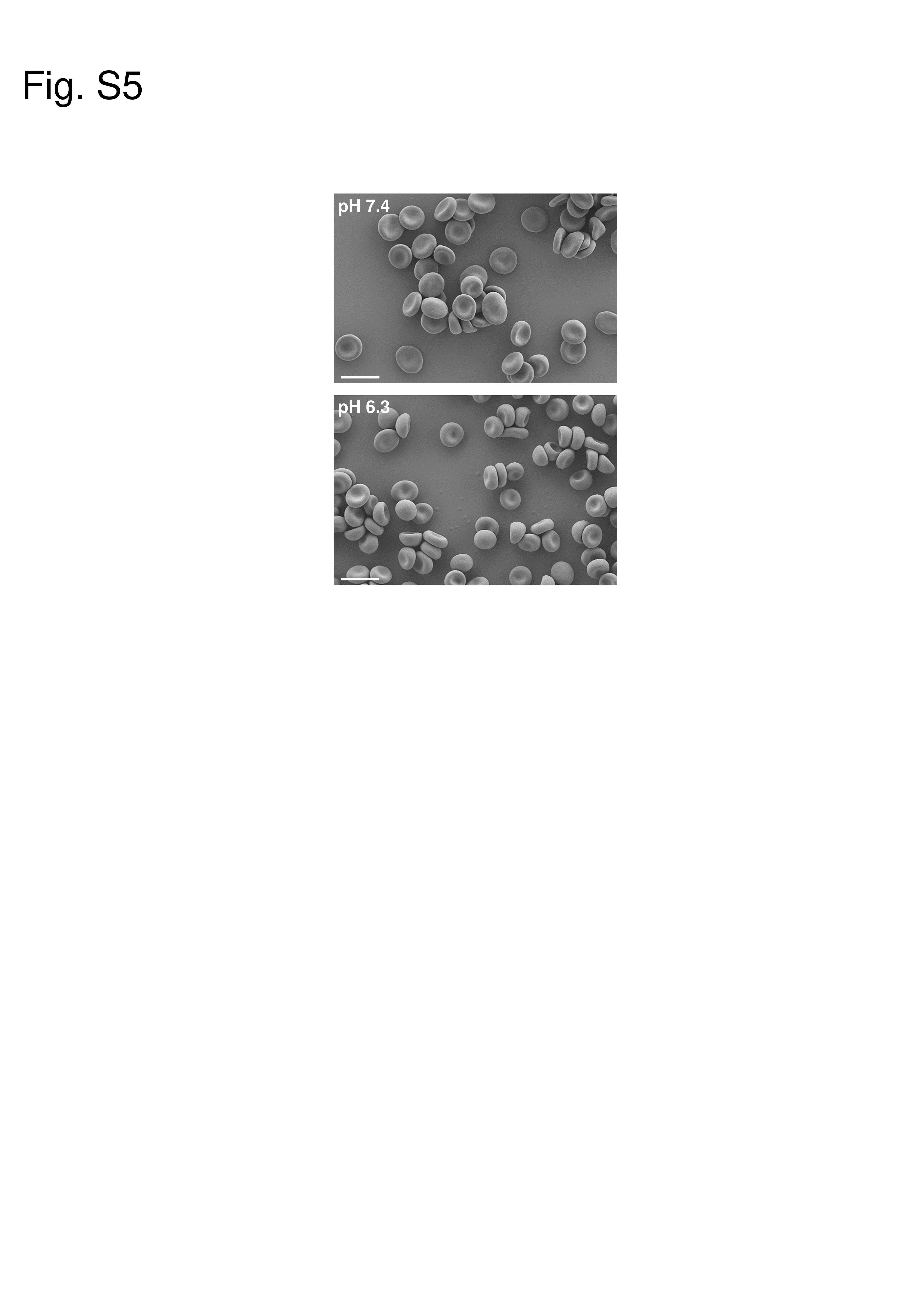

Supplement: S5 Fig — Scanning electron microscopy of RBCs exposed to pH 7.4 or 6.3 for 2h. RBCs were fixed with 1% glutaraldehyde, dehydrated by subsequent treatment with increasing concentrations of ethanol. Specimen were mounted and analyzed on a scanning electron microscope (Zeiss) with a 100’000-fold magnification. Bar, 10 μm. (TIF) [file ppat.1009434.s005.tif]

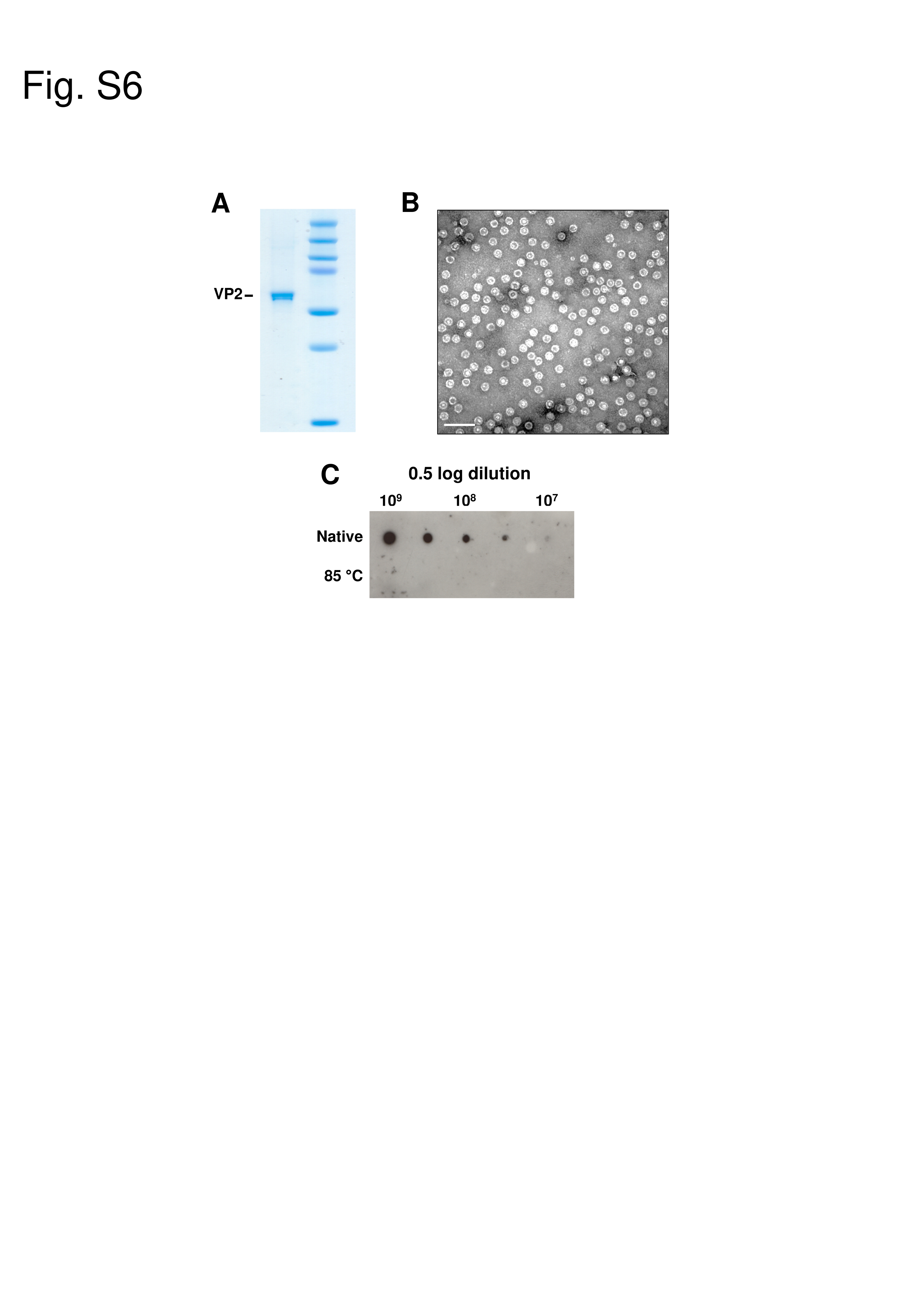

Supplement: S6 Fig — (A) Capsid protein purity of VLPs (VP2-only particles) was verified by SDS-PAGE. Capsid integrity was analyzed by electron microscopy (B), and by dot blot hybridization with an antibody against intact capsids (860-55D) (C). Bar; 100 μm. (TIF) [file ppat.1009434.s006.tif]

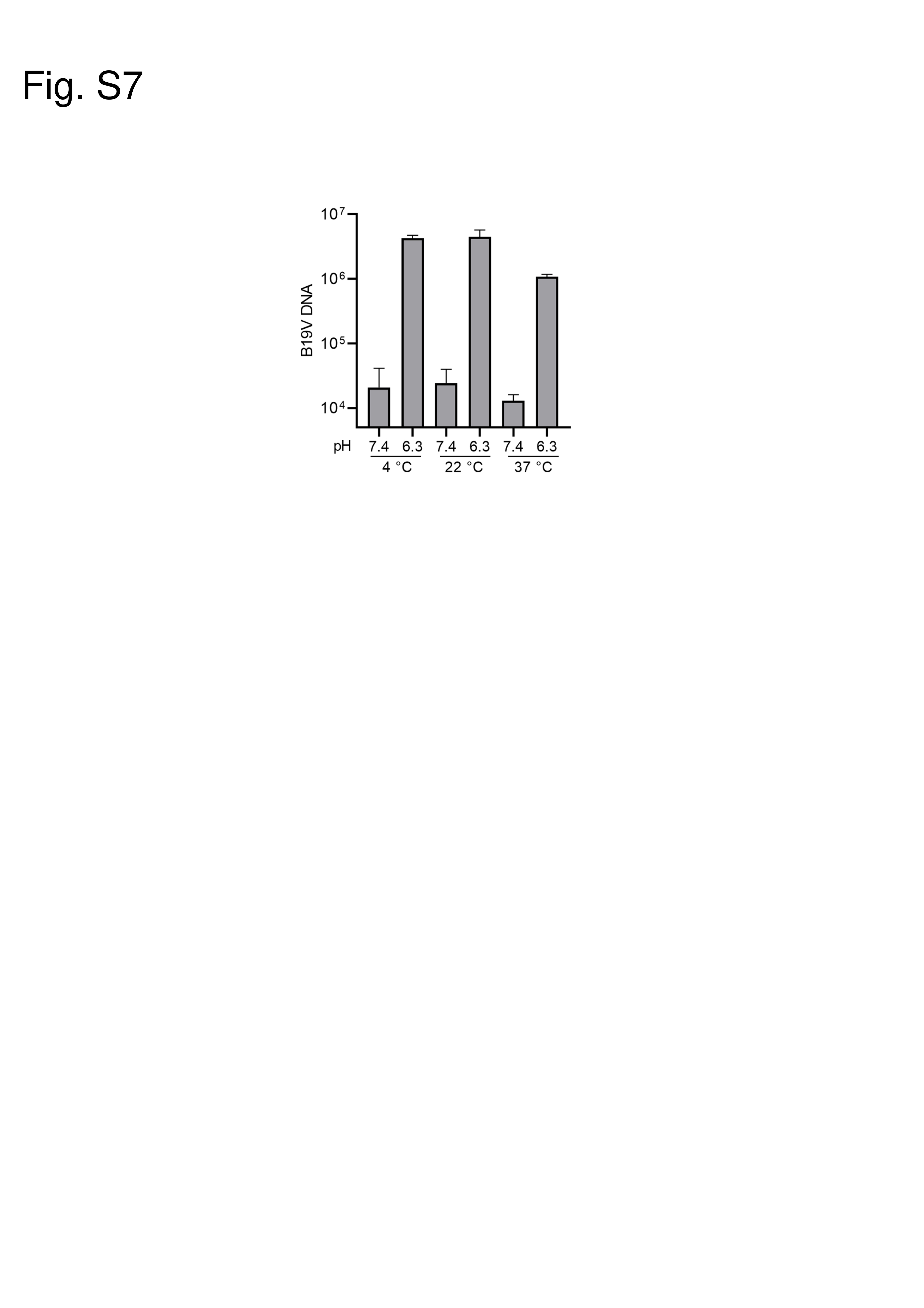

Supplement: S7 Fig — RBCs (0.5% in 100 μl PiBS) were incubated with B19V (5x10^9^) at pH 7.4 or 6.3 at different temperatures for 1h. The cells were subsequently washed at room temperature or at 4°C and viral DNA was extracted and quantified by qPCR. (TIF) [file ppat.1009434.s007.tif]

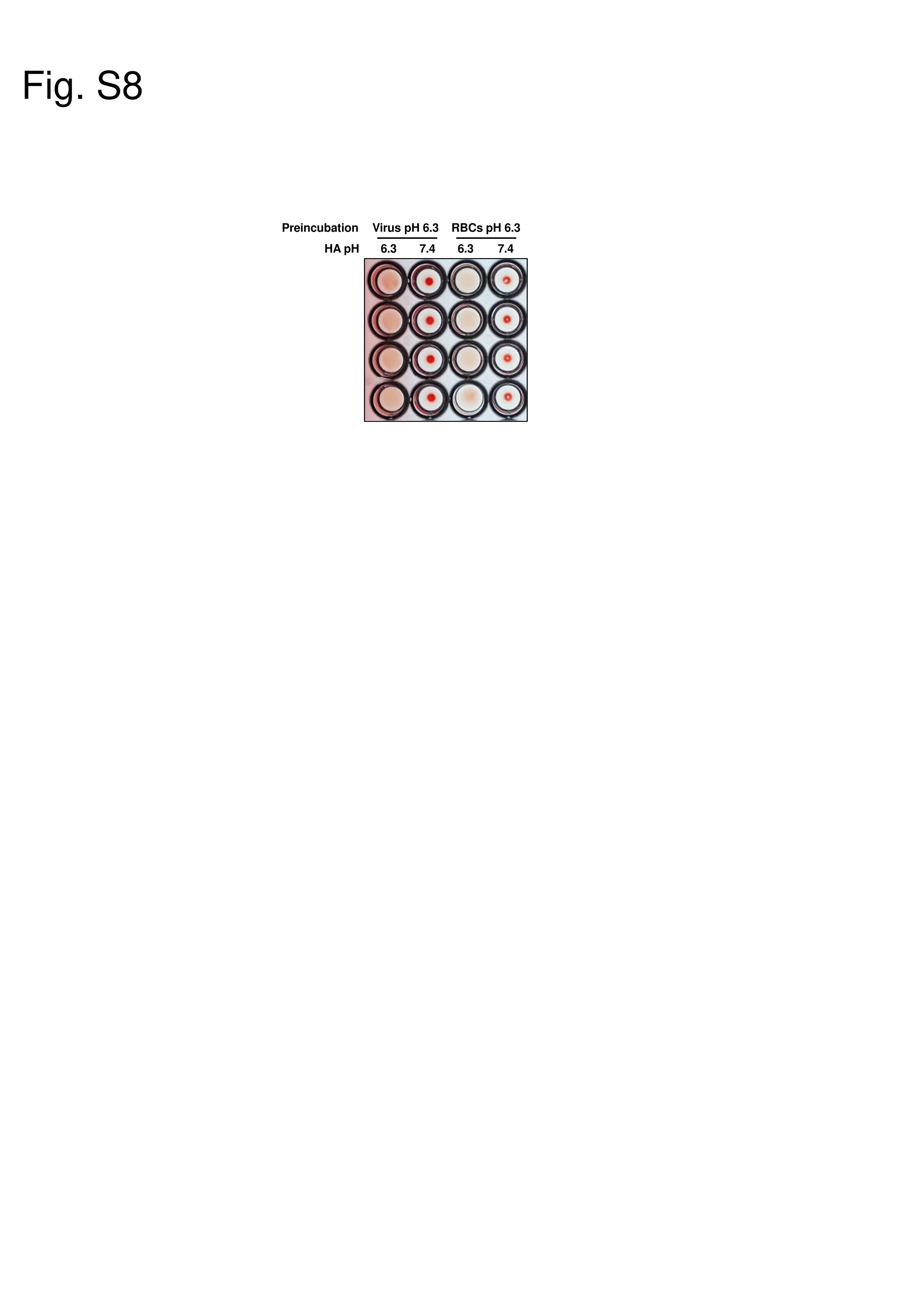

Supplement: S8 Fig — B19V (5x10^9^) and RBCs (0.5% in 100 μl PiBS) were incubated separately at acidic pH for 1h. Subsequently, the HA was performed at neutral (7.4) of acidic (6.3) pH. HA, hemagglutination assay. (TIF) [file ppat.1009434.s008.tif]
