# Supplementary material for: Human parvovirus B19 interacts with globoside under acidic conditions as an essential step in endocytic trafficking
Source: PLoS Pathog. 2021 Apr 20;17(4):e1009434. doi: 10.1371/journal.ppat.1009434 (PMC8087101; doi:10.1371/journal.ppat.1009434)
Supplement: Attachment — Submitted filename: Response to reviewers.docx [file ppat.1009434.s009.docx]

**Reviewer's Responses to Questions**

**Part I - Summary**
Please use this section to discuss strengths/weaknesses of study, novelty/significance, general execution and scholarship.

Reviewer #1: The manuscript “Parvo B19 interacts with globoside under acidic conditions to facilitate endocytic trafficking” by Bieri, Leisi, Bircher and Ros is aa comprehensive and elegant study that redefines the role of Gb4 in infection of cells by B19. Gb4 was previously thought to be the principle cell surface receptor for B19 based on virus binding to Gb4 and the fact that Gb4 knockouts are resistant to infection. The broad cellular distribution of Gb4 however is counter to the restricted tropism of the virus. The authors show that rather than playing a role in initial binding to cells that Gb4 plays a fundamental role downstream of initial entry to facilitate endocytic trafficking of the viral particle and its genome to the nucleus to initiate infection. Another cell surface molecule identified only as VPu-Receptor is the major cell surface receptor for the virus and its expression is highly restricted to erythroid progenitor cells (EPC). This paper focuses on the role of Gb4, confirms its interaction with B19 capsids, and defines the cellular requirements that dictate those interactions.

In summary this is an elegant and comprehensive paper, the data are clean and convincing, and certainly support their major conclusions.

Reviewer #2: Bieri and colleagues provide compelling evidence that a specific sphingoglycoprotein, designated Gb4, is a host factor for parvovirus B19 entry into erythroid progenitor cells. Although the principal attachment receptor for this virus has yet to be identified, it appears that cell tropism is largely determined by recognition of Gb4 that presumably interacts with the VP2 capsid protein. The authors show that this interaction does not occur at neutral pH, thereby explaining how the broad distribution of Gb4 does not result in infection of non-productive host cells. Rather the Gb4-B19 virus interaction occurs at a reduced pH, similar to that found in endocytic vesicles. Abrogation of Gb4 in cells deficient of this molecule results in efficient virus attachment and internalization, however the virus fails to progress and remains trapped in vesicles. The results are quite clear as presented and add a new dimension to our understanding of how multiple host cell molecules facilitate virus entry. I have only a few suggestions for the authors to consider in order to clarify a few sections of the paper.

1. The authors could bolster the Discussion a bit with regards to the identity of the unknown attachment receptor. For example, do they know the relative binding affinity for the putative receptor vs that for Gb4?

The differences in affinity between the VP1uR and Gb4 cannot be determined accurately, since they operate under different conditions, one requiring acidic pH while the other does not. Moreover, the binding affinity to Gb4 increases as pH decreases and therefore results would strongly depend on the pH conditions. Importantly, Gb4 and VP1uR do not compete under natural conditions, VP1uR interaction occurs on the plasma membrane while Gb4 interaction ensues inside the endosomes. Comparison of the affinity of the virus for these two unrelated molecules has questionable scientific value.

2. Figure 5C. I cannot find a description of the results of this experiment in the text. I think it may have been omitted accidentally by the authors.

Figure 5C was mistakenly labeled as Figure 5B. Corrections have been made (Line 295).

3. Why doesn’t the interaction of the virus with Gb4 at low pH result in capsid protein production given that NS1 mRNA is detected at a normal level as shown in Figure 6D.

It seems that the reviewer misunderstood this figure.

When VP1uR is not blocked (ΔN29) and regardless the pH conditions, NS1 mRNA levels and viral capsid production are not affected (Figure 6D and 6E). However, when the VP1uR is blocked (ΔC126), NS1 mRNA levels are reduced to background levels and, expectedly, no capsid progeny is produced.

4. Line 248. I suggest a modification of the title as follows: The early endosomal compartment provides optimal conditions for Gb4 interaction.

The heading in question: “Different to the extracellular environment, the early endosomal compartment offers optimal conditions for Gb4 interaction”

It is generally believed that the extracellular conditions are optimal for Gb4-B19V interaction, as should be expected for a molecule that is considered the primary receptor of the virus. We believe that it is important to keep in the title the information that the extracellular environment is not appropriate for the binding, which is one of the key findings of this study. However, we agree with the reviewer that the word “provides” is more suitable that “offers” and has been changed accordingly.

5. The Material and Methods are lacking in sufficient detail in a few places:

We agree and have added additional information concerning MVM in the Material and Methods that we consider important (Line 471).

Line 469. Please specify the source of the B19 used in this study (is this a clinical specimen or one purchased, etc)?

This virus cannot be propagated in cell culture to high titers. Accordingly, native infectious B19V always originate from a viremic plasma sample. This was specified in the following sentence “B19V-infected plasma sample was obtained from a donation center (CSL Behring AG, Charlotte, NC)…” (line 467).

Line 513. Please give more details of the confocal microscopy.

The microscope used is an LSM 880 (Zeiss, Germany). We have included this information in the revised manuscript (line 514).

Line 535. How was viral NS1 mRNA quantified and what primers may have been used?

The viral NS1 mRNA was quantified in the same manner as described under the section “Transfection”. We have modified the text for clarity (Line 540).

Reviewer #3: The manuscript by Bieri et al., describes, in a comprehensive set of experiments, the role of globoside in the life cycle of human parvovirus B19. This laboratory has made seminal contributions in the past relating to B19 biology, and the current studies are a fine example of their thoughtful rationale, careful planning of experiments, and lucid presentation of the data. Overall, this is a well-written manuscript in which the quality of the data presented is good, and the conclusions drawn are generally supported by the data shown.

**Part II – Major Issues: Key Experiments Required for Acceptance**
Please use this section to detail the key new experiments or modifications of existing experiments that should be absolutely required to validate study conclusions.

Generally, there should be no more than 3 such required experiments or major modifications for a "Major Revision" recommendation. If more than 3 experiments are necessary to validate the study conclusions, then you are encouraged to recommend "Reject".

Reviewer #1: None

Reviewer #2: (No Response)

Reviewer #3: None.

**Part III – Minor Issues: Editorial and Data Presentation Modifications**
Please use this section for editorial suggestions as well as relatively minor modifications of existing data that would enhance clarity.

Reviewer #1: The abstract on the manuscript submission form differs slightly from the abstract in the manuscript and the former has some confusing typos such as:

1st sentence: The glycosphingolipid (GSL) globoside (Gb4) this study, we applied artificial viral
particles, genetically modified cells, and specific competitors to address the interplay
between the virus and the GSL.

Part of a sentence was inadvertently removed from the abstract that appears in the submission form. However, the abstract of the manuscript is complete.

Figure 1A and S2 don’t seem to fit with the results:

“The expression of VP1uR in cells was sufficient to trigger virus attachment and uptake, however, in the absence of Gb4, the intracellular capsids failed to initiate the infection (Figs 1A and S2)”.

These figures are not showing failure to infect…1A is detection of receptor in GB4 KOs and S2 is showing cell cycle arrest. Please clarify.

The reviewer has not noticed that besides the expression of the receptors, Fig. 1A (last panel to the right) also shows production of capsid progeny (or lack thereof in Gb4 KO cells). Figure S2 also deals with infection. Cell cycle arrest has been shown to be a hallmark of successful B19V infection (Luo et al. J Virol 2013, DOI: [10.1128/JVI.02333-13](https://doi.org/10.1128/jvi.02333-13)).

Reviewer #2: Include a description of the studies shown in FIgure 5C.

See comment above concerning wrongful labeling of Figure 5C (line 295). The detailed description of this figure spans lines 287 to 294.

Reviewer #3: The authors may wish to address the following points, which may be helpful to the reader:

1. While it is known that exposure to low pH alone is sufficient for the structural change observed in the adenovirus capsid, previous studies with AAV have suggested that exposure to low pH is necessary but not sufficient (J. Virol., 75: 4080-4090, 2001). Since B19 would also be expected to encounter similar intracellular milieu, in addition to pH changes, which may not directly mimic the in vitro experimental conditions.

While it may be tempting to speculate about the possible influence of early endosomal conditions on the interaction of the virus with Gb4, this is not the focus of this study and we would like to stay grounded to our results. We prefer to avoid comparisons with AAV, since this virus has important differences with B19V. Notably, AAV requires low pH to expose the internal VP1u, while B19V exposes VP1u already at the plasma membrane. However, we agree with the reviewer that it is important to mention the possible influence of early endosomal factors, other than the low pH.

Accordingly, we have modified the following sentence:

Line 447. « Further research will aim to characterize changes in membrane properties mediated by the interaction of the virus with GSL-enriched areas of the endosomal membrane and the influence of additional intracellular factors in the interplay between B19V and Gb4.»

2. Parvovirus B19 binding to RBCs in vitro may also not truly mimic a natural infection in vivo. Weigel-Kelley et al, have proposed that human parvovirus B19 binds to mature erythrocytes, which facilitate trafficking of the virus to the bone marrow where the target cells reside (Blood, 102: 3927-3933, 2003). This reference should be cited.

In our study, the interaction of B19V with RBCs was tested in freshly collected whole blood with the intention to mimic natural conditions. In contrast, in the study of Weigel-Kelley, the interaction of B19V with RBCs was tested (only by immunofluorescence microscopy) with washed RBCs in cell culture media (IMDM), neglecting the natural environment of the blood. Consequently, the inhibitor(s) of B19V-Gb4 interaction, which are associated to the blood plasma, were not present. For this reason, this reference was not cited in this study.
